# The impact of land use and season on the riverine transport of mercury into the marine coastal zone

**DOI:** 10.1007/s10661-014-3950-z

**Published:** 2014-08-02

**Authors:** Dominika Saniewska, Magdalena Bełdowska, Jacek Bełdowski, Michał Saniewski, Marta Szubska, Andrzej Romanowski, Lucyna Falkowska

**Affiliations:** 1 Institute of Oceanography, University of Gdansk, Al. Pilsudskiego 46, 81-378 Gdynia, Poland; 2 Institute of Oceanology, Polish Academy of Sciences, ul. Powstancow Warszawy 55a, 81-712 Sopot, Poland; 3 Maritime Branch, Institute of Meteorology and Water Management, National Research Institute, ul. Waszyngtona 42, 81-342 Gdynia, Poland; 4 Maritime Institute, ul. Długi Targ , 80-830 Gdańsk, Poland

**Keywords:** Mercury, River, Catchment, Storm water, Runoff water

## Abstract

In Mediterranean seas and coastal zones, rivers can be the main source of mercury (Hg). Catchment management therefore affects the load of Hg reaching the sea with surface runoff. The major freshwater inflows to the Baltic Sea consist of large rivers. However, their systems are complex and identification of factors affecting the outflow of Hg from its catchments is difficult. For this reason, a study into the impact of watershed land use and season on mercury biogeochemistry and transport in rivers was performed along two small rivers which may be considered typical of the southern Baltic region. Neither of these rivers are currently impacted by industrial effluents, thus allowing assessment of the influence of catchment terrain and season on Hg geochemistry. The study was performed between June 2008 and May 2009 at 13 sampling points situated at different terrain types within the catchments (forest, wetland, agriculture and urban). Hg analyses were conducted by CVAFS. Arable land erosion was found to be an important source of Hg to the aquatic system, similar to urban areas. Furthermore, inflows of untreated storm water discharge resulted in a fivefold increase of Hg concentration in the rivers. The highest Hg concentration in the urban runoff was observed with the greatest amount of precipitation during summer. Moderate rainfalls enhance the inflow of bioavailable dissolved mercury into water bodies. Despite the lack of industrial effluents entering the rivers directly, the sub-catchments with anthropogenic land use were important sources of Hg in the rivers. This was caused by elution of metal, deposited in soils over the past decades, into the rivers. The obtained results are especially important in the light of recent environmental conscience regulations, enforcing the decrease of pollution by Baltic countries.

## Introduction

For many years mercury (Hg) has been recognised as one of the most dangerous anthropogenic pollutants. Particularly sensitive to Hg contamination is the aquatic environment, where the metal bioaccumulates and biomagnificates with increasing trophic level. Consequently, Hg concentration in tissues of fish, birds and water mammals can be even 10,000 times higher than in the surrounding water (Boening 2000; Schurz et al. 2000). The most common risk of Hg poisoning to humans is through the consumption of fish and seafood. Therefore, gaining an understanding of Hg cycling in terrestrial and aquatic environments is of fundamental importance, especially in areas where the consumption of seafood is common (Gerstenberger 2004).

On a global scale, the main pathway of Hg transport between land and sea is the atmosphere, whereas the load of Hg introduced to seas by rivers is relatively small. In coastal zones, however, rivers can represent the main source of pollutants into water and the sediment (Damart et al. 2013). Only a fraction of riverine Hg reaches offshore waters as most of it tends to be accumulated in the coastal zone, close to the river mouth. Hence, terrestrial Hg is considered to be a significant pollutant of gulfs and bays, where Hg concentrations may be several times higher than those recorded in offshore waters (HELCOM 2010; Horvat et al. 2003; Laurier et al. 2004; Saniewska et al. 2010). This is very important because of intensive phytoplankton production in the river mouth (Wielgat-Rychert et al. 2013).

Hg deposited on terrain with precipitation can be bound by both minerals and organic matter present in the soil, resulting in Hg retention in the soil (Grigal 2002). In regions where the catchment is undisturbed, Hg retention can reach 90 % (Babiarz et al. 2012; Grigal 2002; Scheuhammer et al. 2012). Disturbance of the natural soil profile, through land use change or coverage of the natural terrain with impermeable surfaces such as concrete or tarmac, can significantly affect Hg mobility, and its retention in such areas can be as low as 45 % (Balogh et al. 1998; Eckley and Branfireun 2008; Shanley et al. 2008; Warner et al. 2005). Deposited atmospheric Hg, depending on the type of catchment, can undergo a range of transformations. Land use can therefore be said to significantly affect both the transport of Hg to rivers and its speciation.

Despite a significant decrease in anthropogenic emissions discharged directly into rivers (due to the development of sewage systems and the building of new industrial and municipal sewage treatment plants) and a simultaneous decrease in atmospheric emissions, no significant decreasing trend of riverine Hg loads into the Baltic Sea has been observed (HELCOM 2010). One of the reasons for this is the expansion of municipal infrastructure and subsequent increase of impermeable surfaces, which short-circuit hydrological pathways in stream basins and thus reduce retention (increase yield) of atmospherically derived Hg (Bełdowska et al. 2006; Hławiczka et al. 2003; Lyons et al. 2006). This in turn enhances the rate of Hg elution from land, in the form of surface runoff, thereby causing greater amounts of the element to be transported to the sea.

European Union countries are obliged to reduce emissions of Hg into the environment, and it is therefore important to take into account the outflow of Hg from catchments. Despite the reduction of anthropogenic Hg emissions, increased concentrations of the metal have been observed in the coastal zone at certain times. This suggests that catchment management can significantly impact on Hg circulation in the environment. Large rivers are responsible for the majority of freshwater flowing into the Baltic Sea, but as their systems are complex, it is hard to identify the particular factors which affect the outflow of Hg from their catchments. Taking this into consideration, it was decided to conduct the study using two of the smaller rivers in the vicinity. The goal of this study was to estimate the amount of mercury retained in the catchments of the two rivers and assess the factors affecting land-river transport of Hg. This included ascertaining the influence of catchment type and season on Hg transport and chemistry in small boreal rivers not impacted by heavy industry. In the southern Baltic region, such studies have not been conducted. Thus, the information obtained is essential for assessment of the influence of river management on Hg fluxes to the coastal water of the southern Baltic Sea and the sustainable development of this region. To achieve this goal, the concentration and speciation of Hg were ascertained for water samples collected at different terrain types (forest, wetland, agriculture and urban) within the catchments of these two rivers, both of which are situated in northern Poland. These data, together with water discharge and Hg atmospheric deposition, were used to estimate the flux of Hg into the coastal zone of the southern Baltic Sea (Poland). With heavy rains and flooding becoming more frequent in this part of Europe as a result of climate changes, such information must be considered of importance.

## Material and methods

### Study area

**Fig. 1:**
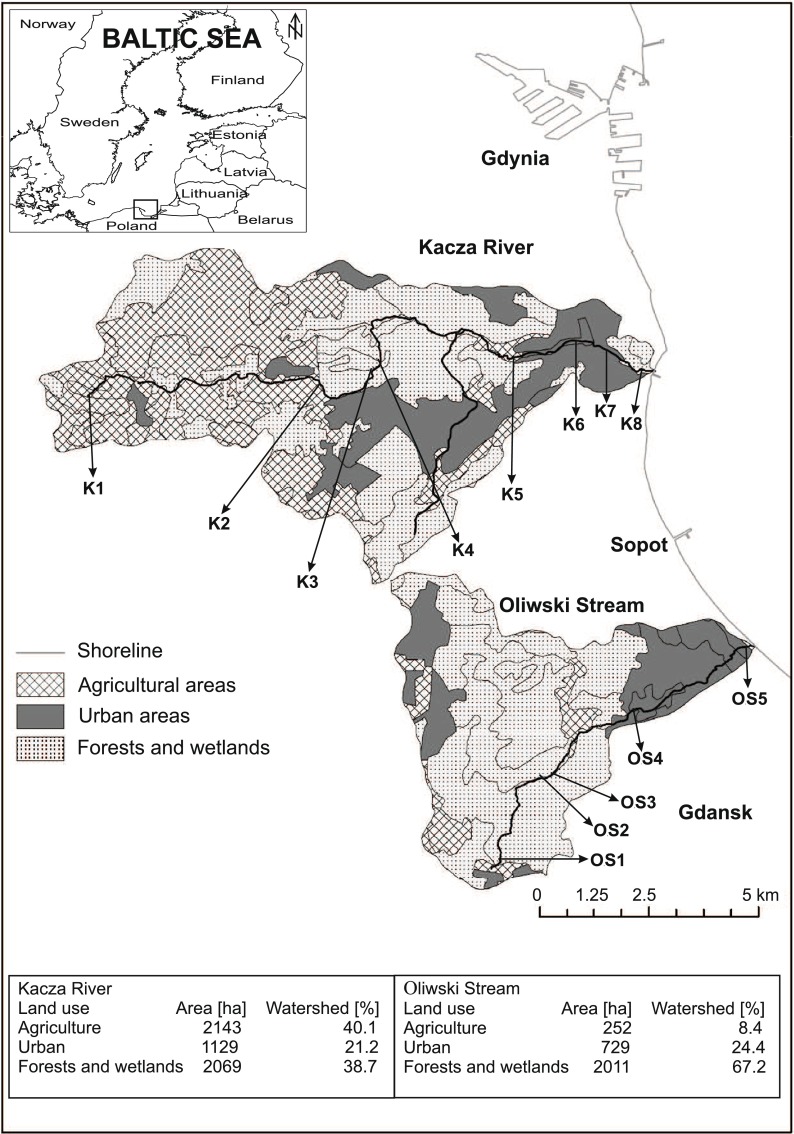
Location of the study area

Water samples were collected from two small urbanised catchments in northern Poland (Fig. 1). Both rivers collect drainage from the metropolitan Tricity (Gdansk, Gdynia and Sopot), which has a total area of 415 km^2^ and a population of almost 750,000 (GUS 2012). Both river catchments have different land use patterns but may be considered typical of the southern Baltic region. Numerous industrial plants (e.g. power plants, municipal waste incinerators, cement and paint plants, a refinery and shipyards) located in the Tricity region introduce pollutants to the environment through atmospheric deposition. However, wastewater from industrial plants does not flow into the studied rivers, and this enabled the influence of catchment terrain and season on Hg biogeochemistry and transport in the rivers to be properly assessed.

The Kacza River has a length of 14.8 km, a catchment area amounting to 53.8 km^2^ and a mean flow in the mouth of 0.2 m s^−1^. It runs through forest, small patches of wetlands and peat bogs, and downriver, it passes through a city of 250,000 inhabitants (Fig. 1). Eight sampling points were designated along the river, the first being close to the springs (K1) in the village of Bojano. The river originates at wetlands situated at 157 to 190 m AMSL and flows through farm fields (K2) towards a forest. In the forest, it flows under a major highway, where stations K3 (ca. 100 m before the highway) and K4 (100 m after the highway) were located. The river then enters a nature reserve area, at the border of which station K5 was located; flows through allotment gardens (K6), under a railway line and main street (K7—100 m after the street); and discharges into the Gulf of Gdansk at Orlowo beach (K8—100 m from the mouth).

The Oliwski Stream has a length of 9.9 km, a catchment area covering 28.7 km^2^ and a mean flow in the mouth of 0.2 m s^−1^. In its upper part, it passes under a major highway (OS1—ca. 100 m downstream from the highway) and then flows through a forest (OS2) and wetland (OS3). The stream then enters urbanised areas and flows under a main street (OS4—50 m downstream from the street) before discharging into the Gulf of Gdansk (OS5—200 m upstream from the mouth) (Fig. 1).

### Sampling

Samples of subsurface water were collected every month between June 2008 and May 2009 from 13 stations at two rivers (eight at Kacza River and five at Oliwski Stream) with the use of an acid-cleaned polypropylene sampler. All samples for mercury analysis will be taken in triplicate. Samples of storm water (*n* = 7 in triplicate) discharged into the Gulf of Gdansk were collected in a similar way.

Water samples for the determination of Hg_tot_ and dissolved gaseous mercury (DGM) concentration were collected directly into a 30-cm^3^ borosilicate vial with Teflon-lined screw cap. The samples for total mercury determination were acidified using concentrated HNO_3_ (0.5 % of the sample volume). The water was stored at 4 °C until analysis. Water samples for determination of DGM were not preserved and were analysed immediately after returning to the laboratory. Water samples for determination of Hg content in the suspended particulate matter (SPM) were collected into 1-dm^3^ bottles of dark borosilicate glass and were filtered through fired, pre-weighed glass fibre filters of pore size 0.7 μm (Whatman). Filtration was carried out immediately after returning to the laboratory, under a laminar flow hood (HEPA class 100). Filters were freeze-dried and then stored at −20 °C in acid-digested polycarbonate boxes. Field, bottle and procedural blanks were collected during sampling and filtration. All Hg concentrations were blank-corrected.

### Analysis

Water samples for Hg_tot_ analysis were oxidised by adding BrCl and pre-reduced with hydroxylamine hydrochloride solution 1 h prior to analysis by CVAFS (TEKRAN 2600, Canada), according to US EPA method 1631 (U.S. Environmental Protection Agency USEPA 1992, 2000). Quality control procedures for water samples included the use of blanks and water spiked with Hg nitrate within the range of 0.5–25 ng dm^−3^ and produced adequate precision (1 % RSD) and recovery (98–99 %). Quality control procedures (three replicate samples, analysis of reference materials BCR-579—coastal sea water) indicated that the measurement uncertainty was less than 5 %. The method detection limit for Hg_tot_ analysis in water samples was 0.05 ng dm^−3^.

Water samples for DGM (Hg^0^ and DMHg) were transferred into an acid-cleaned borosilicate glass impinger. The sample was extracted by means of a stream of pre-purified air, introduced via a glass frit in the bottom of the impinger, over a period of 100 min. The DGM was analysed using amalgamation coupled with AAS detection (GARDIS 3, Lithuania). The method detection limit for DGM analysis in water samples was 0.005 ng dm^−3^.

Samples for Hg associated with suspended particulate matter (Hg_SPM_) were analysed by AAS (AMA 254, Altec Ltd., Czech Republic), after thermal decomposition in pure oxygen. Quality control procedures (triple repetition, BCR-414; GBW 07314) showed that the average error did not exceed 5 %. The method detection limit for Hg analysis in suspended particulate matter was 5 ng kg^−1^.

Hg_tot_ and Hg_SPM_ concentrations were measured in the same water sample. Colloidal material exhibits an ability to transport Hg in a similar way to the dissolved fraction; therefore, dissolved mercury (Hg_dis_) was estimated as the difference between the Hg_tot_ and Hg_SPM_ concentrations.

### Statistical analysis

Statistical tests were performed using STATISTICA 9 computer software. The normality of all data was assessed by applying the Kolmogorov-Smirnov test. The distribution of results was non-normal, and so non-parametric tests were employed to assess the significance of the differences—the Mann-Whitney *U* test, the Kruskal-Wallis ANOVA test and the post hoc Dunn test. Pearson’s correlation coefficient was used to describe the relationship. A significance level of *α* = 0.05 was used.

## Results and discussion

In the Kacza River, Hg_tot_ concentration ranged from 0.2 to 14.7 ng dm^−3^ (median 2.9 ng dm^−3^), whereas in the Oliwski Stream, it was slightly lower and varied from 0.5 to 9.3 ng dm^−3^ (median 2.8 ng dm^−3^). Mason et al. (1994) have suggested that the global average riverine concentration of total Hg is 5.0 ng dm^−3^. Slightly higher values were recorded in the estuarine areas of the southern Baltic Sea (Saniewska et al. 2010). In both rivers, values between 0 and 5 ng dm^−3^ prevailed, contributing to respective Hg_tot_ concentrations of 85 % and ca. 75 %. Similar values have been reported for non-impacted rivers at the same latitude in the USA (Babiarz et al. 2012; Fitzgibbon et al. 2008). Only 1 % of Hg_tot_ measurements in the Kacza exceeded the 12 ng dm^−3^ USEPA standard for Hg to protect against chronic effects to aquatic life (USEPA 1992), while the corresponding value for the Oliwski was 0 %. This suggests that, despite the presence of anthropogenic Hg sources in the catchment area (especially power plants, cement and paint plants, a refinery and shipyards), there is no direct threat to water quality within the monitored area.

No uniform trends were observed of the studied parameters in either river, and no accumulation of Hg was noted when moving downstream from possible sources. The Kacza River and Oliwski Stream pass through forest, agricultural and urban areas, which influenced both the transformation and distribution of Hg (Eckley and Branfireun 2008; Grigal 2002; Warner et al. 2005). In order to estimate the influence of the catchment terrain and meteorological conditions on the Hg levels in both rivers more precisely, a cluster analysis was performed. The following parameters were included in the analysis: Hg_SPM_, expressed both as concentration per water volume (Hg_SPM-V_) and as concentration per gram of particulate suspended matter (Hg_SPM-M_), Hg_dis_, DGM and SPM. All the data were log-normalised prior to analysis.

### The influence of catchment terrain type on mercury levels in the studied rivers

**Fig. 2:**
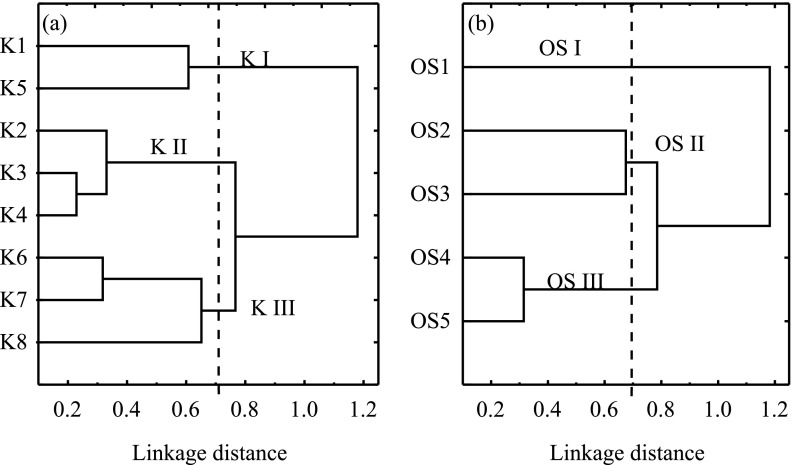
Tree view of cluster analysis showing connections between sampling stations in **a** Kacza River and **b** Oliwski Stream in years 2008/2009

Three types of terrain were distinguished, as a result of cluster analysis, in the catchments of the Kacza River and the Oliwski Stream (Fig. 2). The statistical description of the measured parameters in the distinguished clusters is presented in Table 1. In the Kacza River, the concentrations of all measured parameters differed depending on catchment terrain type with the exception of DGM (Kruskal-Wallis ANOVA test), while in the Oliwski Stream, only SPM did not display variability within the distinguished clusters (Kruskal-Wallis ANOVA test). The types of catchment terrain were significantly different from each other, and it is likely that Hg transformations occurred with varying intensity in each.

**Table 1:** Statistical description of measured parameters in distinguished clusters in the Kacza River (K) and Oliwski Stream (OS) (VI 2008–V 2009)

Estimator	Hg_tot_ (ng dm^−3^)	DGM (pg dm^−3^)	Hg_SPM-M_ (ng g^−1^ d.w.)	Hg_SPM-V_ (ng dm^−3^)	SPM (mg dm^−3^)	Hg_tot_ (ng dm^−3^)	DGM (pg dm^−3^)	Hg_SPM-M_ (ng g^−1^ d.w.)	Hg_SPM-V_ (ng dm^−3^)	SPM (mg dm^−3^)
	Kacza River					Oliwski Stream				
	K I					OS I				
*N*	24	24	24	24	24	12	12	12	12	12
*M*	1.3	34	122	0.6	8.9	6.4	42	264	2.7	10.3
Min–max	0.2–5.5	5–69	30–324	0.2–4.4	1.4–32.6	3.2–9.3	7–90	85–489	0.8–7.1	2.1–55.6
	K II					OS II				
*N*	36	36	36	36	36	24	24	24	24	24
*M*	3.1	24	142	2.0	20.5	2.8	26	110	0.8	8.0
Min–max	1.1–14.7	7–71	61–475	0.8–9.1	6.4–72.0	0.5–5.9	>MDL–60	16–398	0.1–3.6	1.4–37.5
	K III					OS III				
*N*	36	36	36	36	36	24	24	24	24	24
*M*	3.3	33	197	2.1	6.6	2.6	50	231	1.3	4.9
Min–max	0.4–8.4	9–89	66–1,278	0.2–4.8	1.4–29.9	0.6–8.1	10–91	132–1,029	>MDL–6.3	>MDL–20.7

*N* number of measurements, *M* median, *Min*–*max* range

In the Kacza River, the first group (K I) consisted of the least disturbed sites, namely the spring area (K1) and the nature reserve area (K5) (Figs. 1 and 2a). Both sampling sites were located in areas of wetland and meadows uninfluenced by direct anthropogenic pressure, and at these stations, Hg entered the river as a particulate-bound form (Hg_tot_/Hg_SPM-V_ *r* = 0.59, *p* < 0.01). The environmental conditions in these areas, most probably involving the presence of iron compounds eluted from soil to water, encouraged transformation of Hg_SPM_ to dissolved forms and its reduction to DGM (Ababneh et al. 2006; Zhang and Lindberg 2001). Water self-purification processes in these areas were especially intense, judging by the elevated concentrations of DGM (Table 1). This was duly reflected in the negligible contamination of these areas by Hg (median Hg_tot_ 1.3 ng dm^−3^, median Hg_SPM-M_ 122 ng g^−1^ d.w., median Hg_SPM-V_ 0.6 ng dm^−3^; Table 1), providing background values for more contaminated areas downriver (post hoc Dunn test).

Another situation was observed in the spring area of the Oliwski Stream (OS I; Figs. 1 and 2b), where the highest values for the majority of studied parameters were recorded (post hoc Dunn test) (Table 1). This was caused by the discharge of runoff water into the stream from the surfaces of nearby parking lots, roads and supermarkets. Storm water collected in Gdynia at that time was found to be highly polluted by Hg compounds (Table 2), and only the DGM concentration was lower than in river water (Mann-Whitney *U* test). As a result, the Hg_SPM-M_ concentration was two times higher in the spring area of the Oliwski Stream (OS1) than in the Kacza River spring (K1) and the Hg_tot_ concentration was five times higher. Environmental conditions in the forest areas caused the mineral particulate-bound form of Hg, common in runoff water, to be converted to the dissolved form (Hg_tot_/Hg_SPM-M_ *r* = −0.62, *p* = 0.03), as demonstrated by an increase in the Hg_dis_/Hg_tot_ ratio (ca. 62 %) when compared with runoff water (ca. 20 %). This stimulated self-purification processes in the area, as confirmed by the relatively high concentration of DGM (Table 1). In areas away from direct anthropogenic pressure along the Kacza (K I), Hg remained in equilibrium—Hg entering the rivers with surface runoff was partially reemitted into the atmosphere and partially transported to the downstream sections of the river. However, in the case of the Oliwski, when the inflow of pollutants was increased by the discharge of runoff water from urban areas (OS I), natural depuration processes were insufficient to maintain the equilibrium. This resulted in the Oliwski Stream spring (OS I), opposite to similar terrain in the Kacza River (K I), being the most polluted of all the studied areas in terms of Hg (Table 1).

**Table 2:** Statistical description of studied parameters in storm water discharge

Estimator	Hg_tot_ (ng dm^−3^)	DGM (pg dm^−3^)	Hg_SPM-M_ (ng g^−1^ d.w.)	Hg_SPM-V_ (ng dm^−3^)	SPM (mg dm^−3^)
*N*	7	7	7	7	7
*M*	24.3	20	688	18.8	57.1
Min–max	14.7–46.8	5–40	387–1,411	14.4–39.7	9.6–180.2

*N* number of measurements, *M* median, *Min*–*max* range

The cluster analysis-distinguished group (OS II) included points located in forest and wetland, away from anthropogenic influence (OS2 and OS3; Figs. 1 and 2b). The natural catchment terrain efficiently inhibits water runoff and Hg inflow to rivers (Eckley and Branfireun 2008; Warner et al. 2005), and on the Oliwski, this manifested itself in significantly lower Hg_tot_ and Hg_SPM_ concentrations in the OS II area as compared to OS I (post hoc Dunn test) (Table 1). Waters draining wetlands and forests have been found to have higher Hg_dis_/Hg_tot_ ratios compared with other surface waters (Babiarz et al. 2012). Wetlands are additionally thought to be significant sources of methylmercury (MeHg) to rivers (Balogh et al. 2005; Shanley et al. 2008), and this may explain the higher concentrations of Hg_dis_ and MeHg observed in these streams. While natural drainage systems (including forest, wetland and meadow) slow the outflow of water and soil to nearby rivers, they may, however, provide an environment in which Hg transformation is enhanced, resulting in increased Hg_dis_ and MeHg concentrations (Babiarz et al. 2012; Balogh et al. 2005; Grigal 2002).

In the areas (K II) where both farm fields (K2) and forests (K3 and K4) occurred (Figs. 1 and 2a), a particularly important role was probably played by soil erosion and surface runoff (Babiarz et al. 2012; Lyons et al. 2006), especially in arable land (Hg_tot_/Hg_SPM-V_ *r* = 0.84, *p* < 0.01). As a result, these areas demonstrated the highest concentration of SPM among all the studied sites (post hoc Dunn test) (Table 1) which in turn contributed to an increase of Hg_tot_ concentration (Hg_tot_/SPM *r* = 0.61, *p* < 0.01). This supports the suggestion that erosion in agricultural areas is an important source of Hg_SPM_ to aquatic systems (Balogh et al. 1998; 2005; Grigal 2002). Balogh et al. (1998) proved that extended agriculture and artificial drainage systems intensified soil erosion rates and that this led to increased Hg loads entering a studied river, in contrast to areas where natural vegetation prevailed. In farmland-forest areas (K II), the concentration of Hg_tot_ was significantly higher than that of the spring area (K I; Mann-Whitney *U* test) and was comparable to those observed in urban areas (K III) (Table 1). This suggests that, as with urban areas, arable land can also become a significant source of Hg for aquatic systems. The likely cause for this is the accumulation of Hg in the soil, as a result of prolonged agricultural use of plant protection products and seed dressings containing Hg compounds.

The lowest DGM concentrations measured among the described clusters were found for forest/wetland (OS II) and forest/agricultural areas (K II) (Table 1). Rocha et al. (2000) proved that Hg reduction dominates in waters with low organic matter concentration, whereas an increase in organic content (i.e. humic substances) leads to an increase in the concentration of reactive organic matter-mercury complexes and a decreased rate of Hg volatilization (Babiarz et al. 2012; Denkenberger et al. 2012). In addition to this, restricted levels of sunlight reaching the water (full canopy shading) can also inhibit the Hg reduction process, which is highly dependent on solar radiation and temperature (Costa and Liss 2000; Poissant et al. 2004). As a result of these factors, the depuration processes were found to be at their slowest in the forest areas of the studied catchments.

The final sections of the Kacza River (K III: K6–K8) and the Oliwski Stream (OS III: OS4 and OS5) were characterised by dense population and heavy traffic (Figs. 1 and 2). In the latter sections of both rivers, Hg_SPM-M_ concentration was significantly elevated (ca. 30 % in comparison to K II and more than 200 % in comparison to OS II) (post hoc Dunn test) due to SPM characterised by higher Hg concentration than that of the river entering the river water with surface runoff from these urban areas (Hg_SPM-M_/Hg_SPM-V_ Kacza *r* = 0.54, *p* < 0.01; Oliwski *r* = 0.60, *p* = 0.01). This was also confirmed by high Hg concentrations determined in storm water (Table 2). The same urban areas (K III and OS III) were characterised by elevated concentrations of DGM (Table 1). The Hg in these areas was probably bound in weak complexes with mineral SPM, and it is known that SPM containing mineral particles of semiconductor type (i.e. iron, manganese oxides) acts as a major catalyst in the formation of gaseous Hg (Ababneh et al. 2006; Denkenberger et al. 2012; Zhang and Lindberg 2001). However, further studies are needed in order to confirm this phenomenon.

### The influence of meteorological conditions on mercury levels in the studied rivers

Urban areas around the two rivers (K III and OS III) were selected for analysis of how meteorological conditions influence Hg concentration levels. Owing to a lack of significant differences between the parametrical values determined in the urban sections of both rivers (Mann-Whitney *U* test), the datasets were combined. This ensured a number of measurements (*n* = 60) which was sufficient to distinguish key periods for Hg transformations in the course of cluster analysis. In other areas, significant differences between the two rivers (Mann-Whitney *U* test) did not allow for the joining of datasets and the number of measurements was therefore insufficient (*n* < 40) for cluster analysis.

**Fig. 3:**
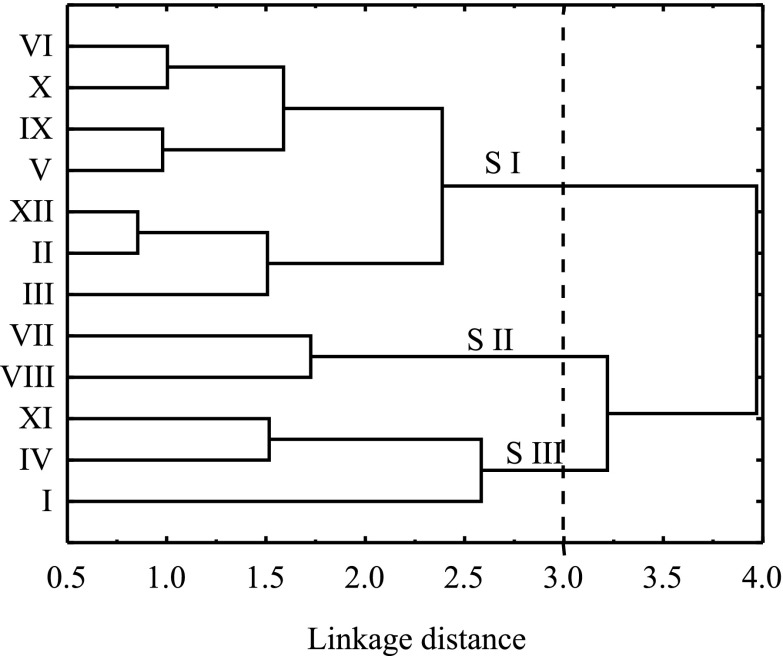
Tree chart of cluster analysis characterising connections between the months of sampling in urban and mouth areas of the Kacza River and Oliwski Stream in the years 2008/2009

In the course of cluster analysis, three periods characterised by different meteorological conditions were distinguished: S I (normal), S II (wet) and S III (dry) (Fig. 3). A statistical description of the studied parameters during these particular periods is given in Table 3. However, significant differences were observed between these groups for Hg_tot_ and Hg_SPM_ (Kruskal-Wallis ANOVA test), suggesting that the concentrations of DGM and SPM were only slightly affected by meteorological and hydrological conditions in the catchment area and that their concentrations were governed by different factors.

**Table 3:** Statistical description of studied parameters in each group in urban and mouth areas of the Kacza River and Oliwski Stream (VI 2008–V 2009)

Estimator	Hg_tot_ (ng dm^−3^)	DGM (pg dm^−3^)	Hg_SPM-M_ (ng g^−1^ d.w.)	Hg_SPM-V_ (ng dm^−3^)	SPM (mg dm^−3^)
S I					
* N*	35	35	35	35	35
* M*	2.9	38	219	1.5	5.9
Min–max	0.6–8.4	10–91	95–663	<MDL–6.2	<MDL–30.0
S II					
* N*	10	10	10	10	10
* M*	3.7	35	571	3.0	4.3
Min–max	2.5–4.4	9–60	166–1,278	0.6–4.0	1.4–7.5
S III					
* N*	15	15	15	15	15
* M*	1.3	34	183	1.0	7.5
Min–max	0.4–4.8	12–60	66–432	0.2–4.5	1.7–23.3

*N* number of measurements, *M* median, *Min*–*max* range

For most of the year (S I: June, September, October and December 2008 and February, March and May 2009), surface runoff played an important role in Hg transformations in both rivers and Hg reaching the rivers was mostly particulate-bound (Hg_SPM-V_/SPM *r* = 0.67, *p* < 0.01). This period was characterised by the largest range of Hg_tot_ concentrations (Table 3), the highest of which were caused by snowmelt (February/March 2009) which transported contaminants accumulated in the snow over the winter (Babiarz et al. 2012; Balogh et al. 2005). Those months were characterised by a total precipitation of between 30 and 65 mm, which amounted to 54 % of the precipitation in the whole study period. Rain water, especially with low pH, accelerates the leaching of metals adsorbed in the soil (Pirrone and Wechmann-Fiebig 2003; Walna et al. 2001). However, in the studied area, acid precipitation is dominant (Siudek et al. 2006) and this could explain the large contribution of Hg_dis_ (average 44 % Hg_tot_) and low Hg content in SPM during that period. Additionally, conditions prevailing in spring (an increase of solar radiation and plankton biomass) and autumn (fast degradation of organic matter) initiated transformation of Hg and the release of reactive Hg into the water column (Ravichandran 2004; Saniewska et al. 2010). The importance of this process must be noted, as it resulted in an increase of the bioavailable form of Hg, which could then be used as a basis for the formation of MeHg.

A different situation was observed during the summer months (S II), which were characterised by the highest total precipitation of 54 and 108 mm for July and August 2008, respectively. In the summer months, frequent downpours (31 % of the sum of precipitation for the whole study period) were responsible for the creation of surface runoff which transported Hg eluted from the ground to the river (Eckley and Branfireun 2008; Vaze and Chiew 2002). Together with a heightened inflow of SPM, and as the result of Hg_tot_ and Hg_SPM_ concentrations in the water which were almost three times higher than during the dry period (S III) (Table 3) (Mann-Whitney *U* test), an increase in the concentration of Hg_SPM-V_ was observed (*r* = 0.53, *p* = 0.04). This indicates that, during the summer months (S II), SPM reaching the river together with surface runoff was enriched with Hg (Hg_SPM-M_/Hg_dis_ *r* = −0.81, *p* = 0.04). Hg in the river occurred mostly in particulate-bound form, amounting to over 90 % of Hg_tot_ (Table 3), and this was probably caused by efficient accumulation of Hg in aquatic organisms, which grow rapidly during the summer season (Bonzongo and Donkor 2003; Ravichandran 2004). As a consequence of this process, a significantly higher Hg_SPM-M_ concentration was observed than in the other distinguished periods (post hoc Dunn test) (Table 3). During this period, Hg that reached the river was immediately incorporated into the food chain.

The third and final group (S III), consisting of the driest months (November 2008, January 2009 and April 2009), was characterised by the smallest land outflow (15 % of total precipitation for the study period). In this period, an accumulation of dust containing contaminants from different sources occurred on the surface of the soil (Eckley and Branfireun 2008) and, subsequently, these contaminants were eluted with rainwater (albeit infrequently) and were usually transported into the rivers in the first flush. As a consequence, a joint increase in SPM inflow and Hg_tot_ concentration was observed (Hg_tot_/SPM *r* = 0.89, *p* < 0.01). However, the limited amount of rain undoubtedly restricted the Hg load to the river, resulting in the lowest Hg_tot_ and Hg_SPM_ concentrations of the entire study period (post hoc Dunn test) (Table 3).

### Input of atmospheric mercury and outflow of mercury from the Kacza River and Oliwski Stream catchments

The wet atmospheric flux of Hg to the Kacza River and Oliwski Stream catchments was estimated to be 2.7 g Hg km^−2^ year^−1^ (Saniewska 2013). Considering Hg concentration in the mouths of the two rivers (K8 and OS5), the average flow of both rivers, 0.2 m^3^ s^−1^, and the catchment areas, the yields of Hg obtained from the catchments were calculated to be at levels of 0.41 g Hg km^2^ year^−1^ for the Kacza and 0.78 g Hg km^2^ year^−1^ for the Oliwski. The outflow from the Kacza River and the Oliwski Stream catchments contributed 15 and 30 % of wet deposition flux in this area, respectively. This indicates that atmospheric Hg retention takes place in the catchments of both rivers and is supported by values which are typical of catchment retention of Hg, generally ranging from 70 to 95 % (Babiarz et al. 2012; Grigal 2002; Lawson and Mason 2001).

## Conclusions

This study was conducted in a relatively small area under no direct industrial impact. The two river catchments exhibit different land use patterns but are typical of the southern Baltic region. This enabled the recognition of Hg transformations and assessment of the influence of the catchments on the form and amount of Hg entering the southern Baltic Sea. In areas where the catchments are anthropogenically undisturbed, Hg remains in equilibrium—Hg entering the rivers with surface runoff was partially reemitted into the atmosphere and partially transported to the downstream sections of the river. As a result, these areas remain unpolluted with Hg. However, the natural drainage systems (including wetland and forest) that slow the outflow of water and soil to the rivers may also provide an environment in which Hg transformation is enhanced, resulting in higher Hg_dis_ concentrations and establishing suitable conditions for the formation of MeHg.

Despite the reduction of anthropogenic Hg emissions, at certain times, increased input of the metal to the coastal zone was observed. Surface runoff from urban areas was identified as the single most important source of Hg found in these rivers, while land erosion and the ability of soil to accumulate metals also indicated farm fields to be an important source. In general, any anthropogenic changes within a catchment (development of arable lands or urban areas with impermeable surfaces) can short-circuit hydrological pathways of Hg in river basins, thus reducing the retention (and increasing the yield) of atmospherically derived Hg. Therefore, an increase in Hg being transported into the two studied rivers may be attributed to increased coverage of arable lands and the diminishing size of undisturbed catchment area, as well as development of urban infrastructure and the discharge of untreated storm water.

The levels of Hg concentration in Kacza River and Oliwski Stream were found to be governed by the intensity of rainfall. In the summer months, characterised by intense rains, Hg_tot_ concentration was observed to increase by up to threefold, in comparison to the period with the minimum amount of precipitation. Additionally, Hg was bound to SPM (probably phytoplankton) most intensely in those months, resulting in concentrations of Hg_SPM_ that were significantly higher than those observed at other times and effecting its immediate incorporation into the riverine food chain. Draught or limited precipitation periods also contributed to Hg accumulation in the catchment areas. The maximum values of Hg_tot_ concentration (>8 ng dm^−3^) resulted from snowmelt transporting Hg which had accumulated in snow over the winter, but moderate rainfalls were also found to enhance the inflow of bioavailable Hg_dis_. The observations presented as part of this study are particularly important for marine life in the coastal zone and estuaries receiving direct river discharge.

Environmental conscience is currently mobilising the Baltic states to reduce Hg loads entering the sea. The results obtained within this study suggest that anthropogenic impact on catchment areas is a major source of Hg in rivers. Catchment management may therefore be considered to have a significant effect on mercury outflow to the sea. While less important on a global scale, it is also essential to determine the impact of land use and season on the riverine transport of mercury into the marine coastal zone. Furthermore, in any calculation of limits for the use and emission of Hg by individual countries, it must be advocated that the outflow of this element from catchment areas (which is particularly large during intense rainfalls, shortly after draught periods or when untreated storm water discharge enters the river) be taken into account.
